# Supplementary material for: A Likelihood Approach for Real-Time Calibration of Stochastic Compartmental Epidemic Models
Source: PLoS Comput Biol. 2017 Jan 17;13(1):e1005257. doi: 10.1371/journal.pcbi.1005257 (PMC5240920; doi:10.1371/journal.pcbi.1005257)
Supplement: S1 File — (TAR.GZ) [file pcbi.1005257.s014.tar.gz › HSPH_Online-SI-Revision/EnKF07/n100-extreme/n100-extreme_EnKF_table4.pdf]

1	R0	{2.76052, 16.3913}	{2.69297, 9.24862}	{{1.86677, 7.31079}, {3., 10.1284}}	{14.0717, 43.5761}	inf	11 577.	6263.49	{348.287, 24 584.3}	92.1696	0.863451	newly	4336.	4646.29	{212., 18 225.}	119.617	0.728226	Reff	0.335762	0.512452	{0.0596474, 1.36704}	119.905	ar	92 090.	169 660.	{148 459., 242 375.}	84.2332
2	R0	{2.18202, 15.1734}	{2.38266, 3.1725}	{{1.37271, 1.21172}, {3., 5.06475}}	{37.2891, 79.0917}	inf	9523.	12 674.7	{2548.88, 45 456.3}	107.027	0.36705	newly	3796.	17 048.8	{4808., 57 991.}	354.412	0.465684	Reff	0.491915	0.639837	{0.366097, 1.08778}	49.0335	ar	84 264.	196 992.	{167 853., 286 563.}	133.779
3	R0	{2.57891, 12.5971}	{2.66829, 7.42579}	{{2.10349, 3.57316}, {3., 8.52691}}	{17.1076, 41.0516}	inf	6810.	4158.95	{259.651, 12 736.7}	92.9968	0.756779	newly	3181.	5250.38	{152., 12 709.}	168.055	0.673246	Reff	0.320791	0.657254	{0.0636955, 2.10347}	161.486	ar	90 302.	173 193.	{149 675., 234 886.}	91.7931
4	R0	{2.5856, 9.28508}	{2.40055, 8.58298}	{{1.8246, 4.25767}, {3., 10.8612}}	{22.8637, 16.5858}	inf	4198.	9017.12	{2306.56, 17 744.9}	133.462	0.2469	newly	2428.	7269.68	{1284., 17 185.}	216.282	0.337031	Reff	0.287622	0.70104	{0.189011, 1.82459}	163.84	ar	90 312.	196 694.	{166 200., 255 939.}	117.793
5	R0	{2.4406, 12.1341}	{2.15063, 7.22328}	{{0.692159, 5.50103}, {3., 7.87114}}	{40.7612, 40.4712}	inf	8911.	4083.23	{489.201, 9398.14}	82.8279	0.697287	newly	4440.	3212.28	{352., 8976.}	89.9311	0.581836	Reff	0.386079	0.414937	{0.128338, 0.726252}	54.0508	ar	88 597.	165 442.	{154 099., 192 116.}	86.7349
6	R0	{2.72155, 15.4094}	{2.66801, 10.099}	{{1.86683, 7.40899}, {3., 11.4768}}	{15.0635, 34.4618}	inf	11 194.	5450.38	{447.719, 13 504.4}	82.048	0.731921	newly	4357.	3746.38	{257., 10 171.}	94.3824	0.615863	Reff	0.34629	0.666755	{0.071676, 1.85873}	127.3	ar	91 682.	167 946.	{146 156., 236 601.}	83.183
7	R0	{2.63781, 14.3935}	{2.74026, 10.045}	{{2.16212, 6.69918}, {3., 17.9607}}	{15.0646, 44.5219}	inf	9306.	6267.88	{690.918, 16 138.1}	78.512	0.568797	newly	3953.	6308.59	{523., 23 413.}	139.473	0.51537	Reff	0.327906	0.906054	{0.0410265, 2.16209}	221.46	ar	91 192.	191 462.	{159 936., 275 667.}	109.954
8	R0	{2.82391, 16.104}	{2.66623, 9.82573}	{{1.8633, 7.36849}, {3., 11.0175}}	{13.5654, 38.9858}	inf	11 648.	4980.99	{541.092, 12 578.6}	78.0109	0.718111	newly	4404.	3451.13	{354., 10 034.}	87.6287	0.582851	Reff	0.322067	0.642368	{0.0772609, 1.70238}	133.422	ar	92 664.	166 770.	{147 389., 224 783.}	79.9733
9	R0	{2.80645, 14.6457}	{2.69146, 12.6635}	{{1.86718, 3.78772}, {3., 19.2579}}	{13.2009, 42.4692}	inf	11 375.	3778.6	{645.489, 8513.71}	69.8282	0.673505	newly	4649.	2186.04	{463., 3671.}	69.3805	0.565256	Reff	0.325885	0.603375	{0.153562, 0.959629}	111.03	ar	92 567.	168 359.	{154 340., 184 988.}	81.8781
10	R0	{1.86213, 5.4271}	{2.53901, 10.5926}	{{2.07031, 7.487}, {3., 16.3052}}	{36.3498, 95.1793}	inf	1169.	11 559.7	{2804.8, 15 008.7}	889.535	0.767745	newly	1101.	7496.5	{1194., 16 363.}	584.799	0.590904	Reff	0.480541	0.59612	{0.167284, 1.4081}	69.2898	ar	75 362.	202 830.	{175 825., 265 499.}	169.141
11	R0	{2.27349, 14.6338}	{2.80172, 5.5718}	{{2.42953, 0.0677875}, {3., 18.2633}}	{23.2342, 73.3194}	inf	9305.	11 527.6	{1939.26, 27 748.2}	114.56	0.435943	newly	3974.	17 065.8	{1106., 66 114.}	366.817	0.458773	Reff	0.44615	0.687509	{0.182113, 2.4295}	106.008	ar	85 805.	194 867.	{163 166., 279 944.}	127.104
12	R0	{2.77261, 14.1894}	{1.9903, 6.97521}	{{0.568677, 3.5323}, {3., 12.3148}}	{37.7292, 50.8421}	inf	10 089.	5158.47	{763.876, 15 057.6}	74.4431	0.613896	newly	4154.	5790.86	{486., 20 264.}	123.013	0.478585	Reff	0.306207	0.573379	{0.120161, 0.962124}	113.04	ar	92 508.	175 906.	{165 639., 194 990.}	90.1527
13	R0	{2.65402, 19.3935}	{2.63764, 3.26207}	{{1.99344, 2.08428}, {3., 4.07702}}	{17.3035, 83.1795}	inf	15 461.	10 303.7	{2844.23, 25 060.4}	72.2472	0.484485	newly	4998.	24 053.2	{4099., 71 816.}	390.758	0.502919	Reff	0.43088	1.04485	{0.0792407, 1.99342}	168.595	ar	91 184.	208 758.	{169 908., 294 369.}	128.941
14	R0	{1.75297, 4.45354}	{2.19317, 7.53167}	{{0.712161, 5.55796}, {3., 8.04194}}	{64.5221, 69.1166}	inf	1073.	2989.31	{287.903, 5522.73}	203.115	0.449531	newly	1153.	2368.78	{189., 4057.}	136.63	0.37408	Reff	0.522297	0.503765	{0.171088, 0.742244}	48.5492	ar	71 263.	168 570.	{159 120., 176 917.}	136.546
15	R0	{1.99928, 7.1501}	{1.97408, 7.12237}	{{0.710701, 5.55571}, {3., 7.99721}}	{57.3211, 14.9121}	inf	2594.	4811.26	{620.158, 4848.63}	116.815	0.248377	newly	1932.	3517.03	{500., 3638.}	121.605	0.284701	Reff	0.446419	0.313015	{0.0862699, 0.536218}	43.8269	ar	79 602.	168 825.	{157 923., 184 395.}	112.086
16	R0	{2.67398, 10.3134}	{2.03044, 7.02303}	{{0.616897, 5.52205}, {3., 7.86684}}	{38.4535, 31.9039}	inf	4744.	4857.37	{679.616, 6377.39}	89.2821	0.421441	newly	2584.	3544.49	{432., 4352.}	93.2248	0.330559	Reff	0.275794	0.34753	{0.0828824, 0.575098}	70.0059	ar	91 289.	171 932.	{161 310., 186 115.}	88.3377
17	R0	{2.44634, 16.3226}	{2.66811, 3.34701}	{{2.04051, 2.96798}, {3., 4.02665}}	{20.3459, 79.4947}	inf	11 754.	8259.55	{883.646, 28 206.2}	74.1617	0.508327	newly	4470.	18 773.7	{1623., 82 353.}	341.264	0.491635	Reff	0.431632	1.00042	{0.592128, 2.04049}	137.233	ar	88 710.	199 307.	{166 504., 301 193.}	124.673
18	R0	{2.02168, 10.1676}	{2.60863, 9.20691}	{{1.22349, 6.55453}, {3., 17.6684}}	{41.0051, 43.4229}	inf	6854.	9248.54	{954.204, 13 269.7}	100.488	0.387463	newly	4020.	8278.43	{587., 10 796.}	157.245	0.416864	Reff	0.515609	0.790533	{0.380113, 1.22108}	64.6392	ar	80 299.	190 028.	{161 940., 254 454.}	136.65
19	R0	{2.89969, 15.009}	{2.6827, 11.9503}	{{1.85327, 4.94461}, {3., 16.8574}}	{11.9803, 30.7621}	inf	11 247.	4067.09	{965.676, 6746.57}	66.3837	0.568141	newly	4512.	2442.19	{552., 3908.}	60.123	0.452644	Reff	0.292405	0.586659	{0.132083, 0.936719}	124.121	ar	93 349.	166 701.	{153 911., 189 631.}	78.5782
20	R0	{2.68933, 7.34928}	{1.89945, 6.76162}	{{0.333483, 3.71859}, {3., 11.0772}}	{41.1537, 43.5576}	inf	2277.	13 140.3	{2143.36, 31 383.2}	485.868	0.603748	newly	1542.	10 454.2	{1624., 30 052.}	585.118	0.666385	Reff	0.246316	0.339489	{0.0226268, 0.5741}	107.891	ar	91 574.	201 855.	{178 445., 270 806.}	120.428
21	R0	{2.15352, 9.28074}	{2.05936, 7.23527}	{{0.697313, 5.47797}, {3., 7.84957}}	{50.7544, 22.0399}	inf	5169.	4888.23	{1287.74, 8065.31}	95.3501	0.461777	newly	3172.	3826.89	{884., 7175.}	106.938	0.41743	Reff	0.421315	0.415134	{0.0913166, 0.745598}	50.868	ar	83 845.	169 796.	{155 296., 209 025.}	102.511
22	R0	{2.21953, 9.04387}	{2.48916, 6.99577}	{{1.33986, 1.88665}, {3., 10.3968}}	{35.0647, 33.49}	inf	4637.	9394.02	{712.36, 17 651.7}	144.21	0.376767	newly	2827.	10 169.1	{481., 35 695.}	291.997	0.433873	Reff	0.397451	0.586516	{0.20836, 1.22644}	70.7807	ar	84 856.	190 356.	{165 073., 232 096.}	124.328
23	R0	{2.21987, 14.5347}	{2.45018, 3.13788}	{{1.33143, 0.698702}, {3., 5.00503}}	{36.7169, 78.4111}	inf	9287.	13 329.9	{5073.86, 45 638.}	89.538	0.266987	newly	3912.	17 516.3	{6082., 48 301.}	352.321	0.526856	Reff	0.465951	0.595327	{0.163548, 1.28636}	83.4187	ar	85 067.	200 378.	{173 499., 279 955.}	135.553
24	R0	{2.84268, 17.9659}	{2.45712, 3.27222}	{{1.39921, 1.30593}, {3., 4.98821}}	{20.8685, 81.7865}	inf	14 057.	14 891.3	{2578.55, 44 773.8}	87.8644	0.438199	newly	4888.	18 282.9	{2835., 46 467.}	290.402	0.516862	Reff	0.351242	0.55774	{0.070927, 0.982218}	92.6144	ar	92 836.	222 474.	{185 087., 304 813.}	139.642
25	R0	{2.52706, 10.3679}	{2.30281, 6.77757}	{{1.26474, 3.39246}, {3., 12.2878}}	{30.209, 44.9994}	inf	5201.	9892.42	{605.882, 32 826.1}	146.187	0.427514	newly	2743.	16 302.2	{523., 68 269.}	541.266	0.558853	Reff	0.316085	0.586812	{0.172041, 1.32612}	103.866	ar	89 623.	186 102.	{158 946., 273 148.}	107.65
26	R0	{2.62835, 19.1088}	{2.76636, 3.24436}	{{2.28637, 2.63383}, {3., 3.76358}}	{13.1314, 83.0216}	inf	14 411.	12 057.6	{1341.92, 46 232.9}	88.0728	0.50056	newly	4701.	24 696.6	{2206., 108 085.}	440.764	0.544348	Reff	0.418354	1.08238	{0.580606, 2.28635}	163.575	ar	90 908.	207 985.	{169 362., 329 286.}	128.787
27	R0	{2.76831, 19.4155}	{2.83188, 10.3007}	{{2.44529, 7.50483}, {3., 14.2726}}	{9.33089, 46.9463}	inf	12 467.	6478.31	{633.702, 12 104.}	89.349	0.767438	newly	3937.	4909.14	{304., 18 920.}	128.161	0.607367	Reff	0.347063	0.703647	{0.158364, 1.53719}	131.707	ar	92 175.	181 322.	{158 844., 269 652.}	96.7152
28	R0	{2.96609, 16.8903}	{2.17173, 7.06946}	{{0.71753, 5.50068}, {3., 7.82818}}	{28.2677, 58.1448}	inf	11 510.	4040.56	{527.702, 4193.21}	88.1148	0.821734	newly	4186.	3033.32	{359., 3403.}	92.5088	0.570745	Reff	0.274037	0.339046	{0.0437323, 0.554913}	65.6487	ar	94 024.	166 414.	{157 120., 170 415.}	76.9913
29	R0	{2.17046, 14.39}	{2.39547, 3.56749}	{{1.407, 2.80634}, {3., 5.26083}}	{37.0252, 75.2085}	inf	10 054.	11 949.6	{910.495, 44 728.8}	103.389	0.460008	newly	4398.	14 713.2	{1235., 47 573.}	256.459	0.456137	Reff	0.504914	0.548739	{0.168832, 1.05478}	54.9906	ar	84 001.	195 667.	{167 972., 279 669.}	132.934
30	R0	{2.88772, 12.4647}	{2.52555, 4.56713}	{{1.8428, 0.186562}, {3., 14.0546}}	{17.1298, 69.9759}	inf	8175.	8270.67	{1037.19, 17 621.7}	85.2523	0.423839	newly	3775.	14 564.	{1773., 31 932.}	316.921	0.444019	Reff	0.263591	0.677509	{0.0792814, 1.84279}	179.419	ar	93 273.	195 687.	{166 070., 260 531.}	109.8
31	R0	{2.46831, 8.14308}	{1.74789, 7.49551}	{{0.510736, 3.74324}, {3., 18.213}}	{49.4349, 38.2303}	inf	3768.	4761.44	{778.753, 6433.1}	64.5374	0.232613	newly	2494.	3903.67	{811., 4944.}	91.5361	0.256634	Reff	0.317474	0.29617	{0.0320878, 0.611339}	66.323	ar	88 762.	175 213.	{164 742., 186 173.}	97.3967
32	R0	{1.93236, 7.38987}	{2.43543, 5.18991}	{{1.59344, 2.8167}, {3., 6.04709}}	{41.3719, 29.7699}	inf	3204.	5416.17	{440.668, 12 949.3}	138.307	0.438872	newly	2353.	7435.16	{461., 16 730.}	273.459	0.447027	Reff	0.482588	0.504393	{0.243828, 0.845714}	42.3817	ar	77 654.	182 915.	{165 149., 230 063.}	135.551
33	R0	{2.82251, 17.6954}	{2.59373, 7.34941}	{{2.1612, 3.57156}, {3., 8.73222}}	{15.7772, 58.4671}	inf	15 003.	2493.3	{170.178, 3813.98}	85.3309	1.0647	newly	5209.	2499.38	{129., 7313.}	81.7117	0.747156	Reff	0.365938	0.639656	{0.047974, 1.79507}	120.596	ar	92 745.	166 246.	{148 857., 216 065.}	79.2507
34	R0	{1.87083, 12.3994}	{2.45365, 3.44438}	{{1.48082, 2.80557}, {3., 5.17367}}	{46.1033, 72.2214}	inf	8229.	10 276.4	{962.901, 44 008.7}	114.913	0.463473	newly	4286.	12 591.4	{1313., 42 679.}	221.608	0.408649	Reff	0.630488	0.626112	{0.317693, 1.11973}	36.9112	ar	76 282.	186 533.	{155 009., 277 518.}	144.531
35	R0	{2.95779, 16.3003}	{2.60226, 7.16005}	{{2.16345, 3.52594}, {3., 8.8383}}	{13.7896, 56.0741}	inf	14 081.	2692.68	{283.003, 3419.95}	86.5759	1.0431	newly	5272.	2658.98	{264., 6670.}	81.8854	0.73349	Reff	0.314472	0.625269	{0.111836, 1.34211}	134.015	ar	93 873.	162 167.	{147 232., 193 863.}	72.7514
36	R0	{2.18718, 14.261}	{2.30049, 5.76851}	{{0.967147, 2.64209}, {3., 19.0078}}	{39.3704, 69.5607}	inf	9571.	4562.62	{1435.95, 17 728.}	90.519	0.786033	newly	4189.	5846.54	{539., 26 566.}	136.473	0.512494	Reff	0.488835	0.659379	{0.201247, 0.981463}	60.1201	ar	84 379.	179 114.	{160 397., 236 801.}	112.273
37	R0	{2.44912, 9.31118}	{2.58771, 11.2178}	{{2.03598, 7.37892}, {3., 16.7152}}	{19.5333, 43.126}	inf	4364.	11 105.3	{2239.38, 14 711.7}	172.623	0.307118	newly	2540.	6479.24	{1397., 10 740.}	176.158	0.321791	Reff	0.332493	0.632146	{0.229767, 1.4636}	105.626	ar	88 442.	207 501.	{180 174., 266 728.}	134.619
38	R0	{2.05949, 5.72514}	{2.5334, 6.05838}	{{2.0312, 0.120115}, {3., 18.7888}}	{23.9173, 96.0914}	inf	1447.	9335.57	{1741.82, 16 122.}	548.754	0.640093	newly	1229.	17 944.8	{1399., 74 383.}	1368.22	0.784381	Reff	0.397749	0.653881	{0.107205, 1.63882}	93.5851	ar	81 587.	208 208.	{179 879., 264 320.}	155.197
39	R0	{1.75505, 8.52404}	{2.75325, 9.37618}	{{2.17751, 6.29513}, {3., 18.3997}}	{56.8761, 43.3245}	inf	4589.	4796.62	{353.381, 7145.24}	92.4842	0.475463	newly	3152.	4703.19	{263., 7196.}	133.748	0.518283	Reff	0.605615	0.883048	{0.357243, 2.17749}	66.1638	ar	70 925.	182 719.	{158 731., 244 309.}	157.622
40	R0	{2.24724, 13.7861}	{2.30183, 6.50794}	{{1.01038, 3.37982}, {3., 12.6045}}	{35.2699, 52.7935}	inf	8230.	6612.76	{739.73, 9248.17}	90.8176	0.534919	newly	3630.	7257.69	{534., 9723.}	156.28	0.404749	Reff	0.451088	0.622875	{0.194191, 1.01335}	67.4265	ar	85 257.	179 766.	{159 531., 232 370.}	110.852
41	R0	{1.77635, 8.62406}	{2.67516, 9.88819}	{{1.88433, 7.40345}, {3., 10.8241}}	{50.5987, 20.8858}	inf	4190.	5469.49	{370.542, 11 897.7}	102.958	0.450311	newly	2830.	3771.78	{218., 11 522.}	118.071	0.512779	Reff	0.557952	0.585607	{0.0679888, 1.76703}	72.9612	ar	72 941.	173 089.	{154 334., 247 126.}	137.3
42	R0	{1.80397, 5.4932}	{2.44652, 10.818}	{{1.52591, 8.43005}, {3., 14.7919}}	{44.0286, 96.9335}	inf	1509.	15 626.1	{770.281, 70 237.7}	951.203	0.713812	newly	1451.	8469.23	{654., 28 778.}	507.506	0.555958	Reff	0.511444	0.528617	{0.0633108, 1.23924}	63.3878	ar	73 232.	206 909.	{171 200., 286 999.}	182.539
43	R0	{2.83096, 16.7077}	{2.79493, 3.12835}	{{2.38108, 1.998}, {3., 3.66224}}	{9.27399, 81.276}	inf	13 176.	15 613.6	{4483.9, 45 542.1}	85.2521	0.335157	newly	4750.	31 668.	{6403., 112 101.}	570.531	0.626876	Reff	0.342093	1.08332	{0.604552, 2.33602}	219.921	ar	92 740.	215 210.	{175 732., 330 291.}	132.057
44	R0	{2.73678, 11.6021}	{2.47512, 4.76621}	{{1.81783, 3.85108}, {3., 6.94047}}	{19.7559, 58.9194}	inf	5400.	11 538.1	{990.259, 27 546.}	160.437	0.382401	newly	2650.	13 141.6	{1321., 49 104.}	415.131	0.527561	Reff	0.263771	0.721928	{0.125409, 1.81781}	204.327	ar	92 003.	206 187.	{171 817., 279 091.}	124.109
45	R0	{2.14235, 6.98601}	{2.15023, 7.2133}	{{0.648143, 5.56227}, {3., 7.93836}}	{50.8744, 15.2446}	inf	1905.	4820.09	{953.399, 3419.87}	172.519	0.319084	newly	1420.	3562.7	{787., 2759.}	170.051	0.312887	Reff	0.389458	0.387162	{0.0910756, 0.577554}	44.4591	ar	82 974.	174 518.	{163 141., 183 692.}	110.329
46	R0	{2.23409, 9.21289}	{2.04287, 7.25859}	{{0.685634, 5.51852}, {3., 7.89265}}	{48.3273, 21.2127}	inf	5803.	4091.63	{756.372, 8085.74}	80.6968	0.519215	newly	3539.	3264.02	{510., 8595.}	89.7496	0.47971	Reff	0.407766	0.44925	{0.106988, 0.704793}	60.3587	ar	85 163.	166 791.	{153 651., 195 549.}	95.8493
47	R0	{2.12258, 8.15265}	{2.51913, 11.0378}	{{1.84043, 8.1829}, {3., 12.3952}}	{23.4818, 35.3894}	inf	3887.	11 716.4	{656.929, 18 719.9}	235.662	0.40327	newly	2681.	6422.7	{506., 8406.}	181.068	0.390447	Reff	0.420441	0.650438	{0.262801, 1.61046}	81.4253	ar	82 690.	205 878.	{173 683., 265 794.}	148.975
48	R0	{2.71771, 10.4636}	{2.54245, 10.9354}	{{2.01054, 7.39372}, {3., 17.3337}}	{16.8357, 40.1322}	inf	6665.	13 891.	{2411.17, 51 909.4}	145.061	0.285437	newly	3585.	9037.5	{1548., 30 912.}	191.93	0.348437	Reff	0.28718	0.727372	{0.238779, 1.90444}	162.87	ar	91 716.	213 204.	{176 204., 291 740.}	132.461
49	R0	{2.44369, 6.66477}	{1.8919, 6.43039}	{{0.226115, 3.71171}, {3., 7.6088}}	{48.9876, 36.3609}	inf	1262.	2794.41	{1059.06, 3260.6}	133.067	0.335203	newly	921.	2219.92	{909., 3469.}	151.066	0.373683	Reff	0.285399	0.307101	{0.041421, 0.523191}	70.2662	ar	88 827.	180 887.	{173 666., 184 203.}	103.639
50	R0	{2.77383, 17.9775}	{2.55944, 3.18682}	{{1.87293, 2.43414}, {3., 3.39743}}	{18.6549, 82.2733}	inf	11 601.	15 143.8	{843.32, 62 145.5}	111.834	0.51896	newly	3992.	21 376.7	{1584., 74 780.}	462.325	0.611566	Reff	0.332083	0.71818	{0.313599, 1.34817}	122.29	ar	92 334.	193 059.	{160 456., 297 601.}	109.088
